# Supplementary material for: Association between Serum Zinc and Toll-like-Receptor- Related Innate Immunity and Infectious Diseases in Well-Nourished Children with a Low Prevalence of Zinc Deficiency: A Prospective Cohort Study
Source: Nutrients. 2022 Dec 19;14(24):5395. doi: 10.3390/nu14245395 (PMC9782999; doi:10.3390/nu14245395)
Supplement: Supplementary file 1 [file nutrients-14-05395-s001.zip › nutrients-2003031-supplementary.pdf]

Supplement Table S1. Linear regression analysis to determine the association between serum zinc concentration and toll-like receptor stimulated cytokine responses at the age of 1 year

	Univariate analysis		Multiviariate analysis	
	(95% CI)	*p*	(95% CI)	*p*
TLR1-2				
TNF-α	0.002(-0.02, 0.02)	0.80	0.02(-0.01, 0.04)	0.14
IL-6	-0.004(-0.02, 0.01)	0.51	0.01(-0.01, 0.02)	0.36
IL-10	-0.01(-0.02, 0.01)	0.29	0.01(-0.01, 0.03)	0.47
TLR3				
TNF-α	0.01(-0.03, 0.06)	0.60	0.04(-0.01,0.09)	0.14
IL-6	-0.01(-0.05, 0.02)	0.47	0.01(-0.02, 0.05)	0.44
IL-10	0.005(-0.02, 0.03)	0.66	0.02(-0.02, 0.05)	0.36
TLR4				
TNF-α	5.68(-0.23,34.49)	0.70	20.09(-18.50, 58.68)	0.30
IL-6	-0.002(-0.01, 0.003)	0.42	0.003(-0.01, 0.01)	0.56
IL-10	-0.005(-0.02, 0.01)	0.44	0.003(-0.02, 0.02)	0.76
TLR7-8				
TNF-α	-0.01(-0.02, 0.01)	0.25	0.01(-0.01, 0.02)	0.26
IL-6	-0.006(-0.01, 0.00)	0.07	0.001(-0.01, 0.01)	0.77
IL-10	-0.02(-0.04, 0.001)	0.07	-0.02(-0.05, 0.01)	0.15
PHA				
TNF-α	0.002(-0.02, 0.02)	0.80	0.002(-0.04, 0.05)	0.94
IL-6	0.002(-0.02, 0.03)	0.88	0.002(-0.03, 0.03)	0.92
IL-10	0.01(-0.01, 0.03)	0.38	0.01(-0.03, 0.04)	0.71

Adjusted for gestational age, gender, birth body weight, mode of delivery, maternal allergy, age of solid food introduction, and breastmilk duration

Supplement Table S2. The generalized estimating equation (GEE) for the change of zinc status during the 5-year follow up among patients with infectious diseases with adjustment of potential confounders

Outcome / Interaction effect	1 y		2 y		3 y		5 y	
	OR (95% CI)	*P* value	OR (95% CI)	*P* value	OR (95% CI)	*P* value	OR (95% CI)	*P* value
Pneumonia	Reference	-	0.91 (0.84-0.99)	0.03	0.92 (0.86-0.99)	0.03	0.91 (0.83-0.99)	0.04
Croup	Reference	-	0.97 (0.91- 1.03)	0.27	0.96 (0.91- 1.02)	0.16	0.99 (0.94 -1.05)	0.70
AOM	Reference	-	1.09 (0.94-1.26)	0.25	1.01 (0.90 – 1.14)	0.89	1.02 (0.90-1.14)	0.81
Enterocolitis	Reference	-	0.84 (0.73-0.93)	<0.001	0.95 (0.86 – 1.06)	0.39	0.91 (0.81 – 1.02)	0.09
UTI	Reference	-	0.98 (0.91 – 1.06)	0.56	0.98 (0.90- 1.05)	0.50	0.97 (0.91- 1.03)	0.30

Adjusted for gestational age, gender, body weight at 1y, mode of delivery, and maternal education
